# Direct pulp capping of primary molars using a novel fast-setting calcium silicate cement: a randomized clinical trial with 12-month follow-up

**DOI:** 10.1080/26415275.2019.1688662

**Published:** 2019-11-13

**Authors:** Ali Vafaei, Niloofar Azima, Leila Erfanparast, Henrik Løvschall, Bahram Ranjkesh

**Affiliations:** a Department of Pediatric Dentistry, School of Dentistry, Tabriz University of Medical Sciences, Tabriz, Iran;; b Department of Dentistry and Oral Health, Aarhus University-Health, Aarhus, Denmark

**Keywords:** Calcium silicate cement, mineral trioxide aggregate, dental pulp capping, deciduous dentition

## Abstract

**Objective:** Novel fast-setting calcium silicate cement with fluoride (Protooth) has been developed for potential applications in tooth crowns. The aim of this study was to evaluate the success rate of direct pulp capping in primary molars using two-layer mineral trioxide aggregate (MTA) and overlying glass ionomer cement versus one-layer novel calcium silicate cement with 4 to 10 minutes setting time.

**Materials and methods:** Ninety bilaterally symmetrical primary molars in the same jaw in 45 patients aged 5 to 7 years were included. Exposed pulps following caries removal were randomly capped with one-layer novel calcium silicate cement or two-layer MTA and glass ionomer cement. All cavities were filled with amalgam. Clinical and radiographic evaluations were performed after six and twelve months. 41 patients were available for the evaluations at the end of the 12-month follow-up.

**Results:** The overall success rate of direct pulp capping, in a split-mouth design, using MTA covered with glass ionomer cement or one-layer novel calcium silicate cement after 12 months were 90% (37 out of 41 cases) and 85% (35 out of 41 cases), respectively, without statistically significant differences after 6 and 12 months.

**Conclusion:** Within the limitations of this study, clinical and radiographic evaluations suggested one-layer novel calcium silicate cement would be successfully used in direct pulp capping of primary molars as a practical alternative to two-layer MTA and overlying glass ionomer cement.

## Introduction

Prevention and treatment of dental caries have a pivotal role in oral health [1]. In this scenario, preserving the vitality of primary teeth with conservative [2,3] or minimally invasive treatment principles [4], such as interim therapeutic restoration approach [5], partial carious removal [6], or atraumatic restorative treatment [7] is essential since pulp infection and the early loss of primary teeth may lead to malocclusion and the risk of esthetic, phonetic, or functional problems [8]. Conservative treatments, such as direct pulp capping, may improve the longevity of primary teeth in the oral cavity until physiologic exfoliation.

Direct pulp cap treatment of exposed pulp includes the application of a capping material over the perforation to maintain the pulpal health and vitality in an attempt to induce a calcified dentin bridge formation [9,10]. The successful outcome of direct pulp capping both in primary teeth [11–13] and the permanent dentition [14] have been reported. Calcium hydroxide-based materials have been used for many decades as the first choice for direct pulp capping [15]. However, the solubility, gradual disintegration [16], low mechanical properties [17], and tunnel-like defects in the majority of the induced tertiary dentinal bridges [18] compromise the long term outcome of direct pulp capping [14].

Use of calcium silicate cement such as mineral trioxide aggregate (MTA) has been discussed as alternative pulp capping materials to overcome the drawbacks of calcium hydroxide-based materials [19]. Calcium silicate cement is able to set in the presence of humidity with release of calcium hydroxide as a hydration byproduct during cement setting [20]. The hydrophilic nature of calcium silicate cements make them a suitable candidate materials in dentistry. Especially in pediatric dentistry, where the establishment of proper isolation is often challenging. Calcium silicate cements are known as excellent biocompatible dental materials [21] with the outstanding sealing ability [22] and antibacterial action [23], which can form apatite precipitations in physiological-like solution [24] and stimulate dentinogenesis in the pulpal tissue [25]. Successful application of MTA in direct pulp capping of primary dentition [12,13] and permanent dentition [19] has been previously reported. Despite the advantages of MTA in the direct pulp capping, the long setting time (165 ± 5 min) [26], difficult handling [27], and wash-out of the material [28] limit its clinical applications, especially in the crowns, where a fast-set cement is needed. The long setting time of MTA in such treatments confronts the clinicians to extend the treatment with a second treatment session or cover the MTA with a second dental material, e.g. a glass ionomer cement in one-session treatment [29].

Recently, a novel calcium silicate cement (Protooth) with fast-setting time and low fluoride release has been developed for practical applications in tooth crown such as cavity base/lining or cementation, in addition to traditional applications of calcium silicate cement in tooth root [30]. The white color novel cement comprises the same components as the original MTA including tricalcium silicate, dicalcium silicate, tricalcium aluminate, and calcium sulfate (calcium-silicate-aluminate composition: CaO 60–70%, SiO_2_ 20–30%, Al_2_O_3_ <5%, tricalcium aluminate >7%, SO_4_ <3%, while other components include fluorides (3.5% wt.), nano-silica, PO_4_ (Patent Pub. No.: WO 2011/023199)) and zirconium oxide (10% wt.) as radiopacifier [30]. The high CaO content with soluble fluorides supports high calcium hydroxide and a low-level fluoride release in the humid environment. The initial mechanical strength of novel calcium silicate cement is superior to that of mineral trioxide aggregate (ProRoot MTA, Dentsply, Tulsa, OK, USA) and tricalcium silicate-based cement (Biodentine, Septodont, St-Maur-des-Fosses Cedex, France) and its long-term mechanical properties increase in the humid environment [30]. The setting time of novel calcium silicate cement is controllable in the range of 4–6 min at clay-like and 8–10 min at fluid consistency [30]. Another version of novel calcium silicate cement called ultrafast novel calcium silicate cement (Ultrafast Protooth) sets in less than 2 min and has been developed for applications, where an ultrafast setting, as in pulp capping, is a need [30].

The *in vitro* cytotoxicity level of the novel calcium silicate cement has been shown to be similar to other calcium silicate-based cements (MTA and Biodentine) and superior to resin-modified glass ionomer cements, zinc phosphate cement, calcium hydroxide-based material, and zinc oxide eugenol-based cement [31]. Acceptable biocompatibility, periapical tissue tolerance, and clinical performance of the novel calcium silicate cement after three years have recently been reported [32]. Novel calcium silicate cement supports apatite formation in physiologic-like solution and the release of fluoride enhances this phenomenon [33]. Interestingly, the apatite precipitations by novel calcium silicate cement close the entrance of dentin-cement gaps, with 50 μm and 300 μm thickness [34], which may contribute to minimize the bacterial leakage.

Altogether, our observations up to now are favoring further clinical investigations on the novel calcium silicate cement. Therefore, the aim of this study was to evaluate the success rate of direct pulp capping using one-layer fast-setting novel calcium silicate cements versus two-layer MTA and overlying glass ionomer cement in primary molars with deep caries lesion. The null hypothesis was that there is no difference in the outcome of direct pulp capping of primary molars using MTA covered with glass ionomer cement versus the novel calcium silicate cement.

## Materials and methods

The study was designed as a 12-month randomized split-mouth clinical trial performed at the Department of Pediatric Dentistry, Tabriz University of Medical Sciences, Tabriz, Iran. The study design was adopted from previous studies by Erfanparast et al. [12] and Asl Aminabadi et al. [11] that evaluated the efficacy of direct pulp capping in primary molars using different calcium silicate-based cements. The research protocol was approved by the respective research ethical committee (reference number: IR.TBZMED.REC.1397.621) at Tabriz University of Medical Sciences, Tabriz, Iran. The study was carried out in accordance with the Helsinki Declaration of Human Rights and was registered at the Iranian Registry of Clinical Trials (IRCT ID:IRCT20100125003168N6). The risks and benefits of the study and implemented treatments were explained thoroughly to the parents and informed consents were obtained from the patient’s parents before any treatment intervention.

The following measures were considered as the patient inclusion criteria:

- Complete physical and mental health.
- No confounding history of the systemic disease or use of local or systemic medicines.
- No history of allergic reactions.

The teeth inclusion criteria for the included patients were:

- Bilateral primary molars with deep cavitated caries lesions (radiographically extending into the pulpal third or quarter of dentin [35]) in occlusal or occlusal and proximal surfaces without pulpal involvement in the initial diagnostic periapical radiograph.
- Teeth with vital pulp, no history of spontaneous pain, pathologic mobility, draining sinus tract, redness or swelling of the vestibule, and sensitivity to palpation and/or percussion.
- No sign of radiolucency in periapical or furcation area, periodontal ligament widening, and evidence of internal or external root resorption in the initial diagnostic periapical radiograph.

### Outcome measure

The primary outcome measure was defined as clinically vital pulp following direct pulp capping. The success of the treatment was defined as a clinically non-symptomatic tooth without periapical or furcal lesion in the radiograph.

### Sample size calculation

The sample size was estimated using previous direct pulp capping studies using MTA [12,19]. Assuming a similar result using novel calcium silicate cement in direct pulp capping of primary molars, the sample size was calculated to be 40 patients with a power of 80% and a two-sided test with an alpha level of 5%. In order to increase the power of the study and taking into account the possible dropouts, 45 patients were included in this study.

### Participants

**Figure 1.**
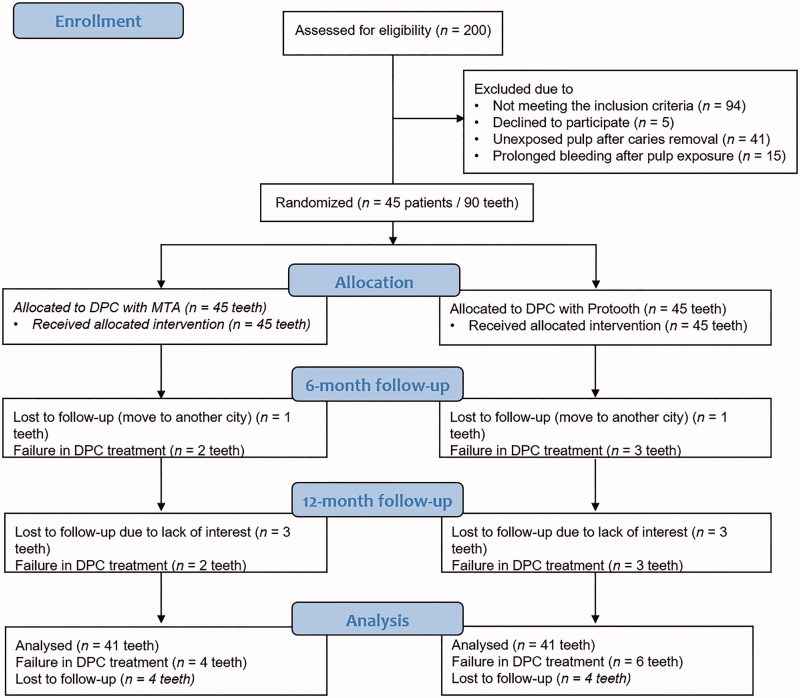
Flow chart of the study from baseline to 12-month follow-up in accordance with Consolidated Standards of Reporting Trials (CONSORT) guidelines. DPC stands for direct pulp capping.

Two-hundred patients, referred from their school to the Department of Pediatric Dentistry to receive an operative dental treatment, underwent routine dental examination. Among these patients, 45 subjects, mean age of 5- to 7-years-old, were included in the study. Figure 1 illustrates the flow diagram of the study. Ninety bilateral primary molars in the same jaw were submitted to direct pulp capping treatment.

### Clinical procedure

In each patient, primary molar with deep caries in the same jaw was randomly allocated to either novel calcium silicate cement group or MTA group. A single expert operator performed the treatments. Clinical and radiographic findings were registered before treatment and after six and twelve months. In the clinical examination, we assessed the spontaneous pain history, increased mobility, presence of sinus tract, vestibule swelling, and tenderness to percussion. In the radiographic evaluation, we assessed the presence of radiolucency in periapical or furcation area, periodontal ligament widening, and evidence of internal or external root resorption. Local anesthesia was administered using 2% lidocaine with 1/80000 epinephrine (Daroupakhsh, Tehran, Iran) using inferior alveolar nerve block for mandibular teeth and local infiltration for maxillary teeth. Isolation was done using a rubber dam. Enamel caries was removed using a diamond fissure bur with a high-speed rotary handpiece under copious water supply. Dentin caries were peripherally removed using tungsten carbide round bur on a low-speed handpiece under water coolant. The cavity was constantly irrigated using 1% NaOCl to wash away the debris under high vacuum in-oral suction. In those cases that pulpal exposure did not happen, the teeth were filled with conventional amalgam and the patient was excluded from the study. In those cases that pulp perforation was observed after excavation, the pulpal exposure area was irrigated with 0.2% chlorhexidine to support the antiseptic procedure. Direct pulp capping treatment was considered according to the following evaluation. The randomization was done by flipping a coin just before pulp capping of the exposed pulp. We performed direct pulp capping if the exposed area was pinpoint (less than one mm), surrounded by sound healthy dentin, and the bleeding was arrested in two to three minutes [12]. Otherwise, the tooth underwent further pulp treatments (i.e. pulpotomy or pulpectomy) and the patient was excluded from the study.

For the direct pulp capping, the cavity was first thoroughly washed with 0.2% chlorhexidine to reduce the bacterial load. The hemostasis was achieved using a gentle pressure of a cotton pellet soaked in sterile normal saline. The cavity was then cautiously dried using a dry cotton pellet. Thereafter, the prepared tooth randomly received direct pulp capping with either one-layer novel calcium silicate cement or two-layer MTA and overlying glass ionomer cement. MTA was prepared according to the manufacturer’s instructions. One gram of the novel calcium silicate cement powder (Protooth, Dentosolve, Aarhus, Denmark) was mixed in a mixing capsule with 195 µl of hydration liquid containing 2% polycarboxylic weak acid as superplasticizer diluted in distilled water. The mixing liquid was precisely pipetted to the capsule and cap-mixed (CapMix; 3 M ESPE, Seefeld, Germany) for 20 s. The operator was not blind to the materials since the manipulation techniques, color, and consistency of the cements were distinguishable. The treatment of the contralateral tooth was done in another session to avoid discomfort by bilateral anesthesia. Novel calcium silicate cement was applied by a round-ended instrument (S Ball Burnisher 27/27S, Premier, USA) with 2 mm thickness over the exposed site and extended approximately one mm peripherally beyond the exposed area. The novel calcium silicate cement was covered with a wet cotton pellet for three minutes to support initial setting. MTA was applied by an MTA carrier (G Hartzell & Son, Concord, CA, USA, #ISS52, 1.8 mm) with 2 mm thickness and extended approximately one mm beyond the exposed site. Then, the material was covered by a second layer of low viscosity glass ionomer cement (Fuji II, GC, Tokyo, Japan) with 2 mm thickness [19]. In both groups, the teeth were immediately restored with conventional amalgam filling (Cinalux, Tehran, Iran). Periapical radiographs were acquired after treatment, 6 months, and 12 months. At each follow-up session, the teeth were clinically and radiographically examined by two expert pedodontists. The two evaluators were blinded to the capping material. The examiners were calibrated before the study in a separate baseline session. The presence of one of the following clinical and/or radiographic findings considered as treatment failure: the presence of spontaneous pain, swelling, sinus tract, sensitivity to percussion internal/external root resorption, widening of the periodontal ligament, and interradicular radiolucency or periapical lesions. Tenderness to percussion and inter-radicular radiolucency were considered as clinical and radiographic criteria, respectively, to assess the interexaminer reliability using kappa agreement coefficient. In case of treatment failure, we performed an extended pulp treatment i.e. pulpectomy and excluded the tooth of patient for study examination in the next follow-up.

### Data analysis

Discrete data including spontaneous pain, sensitivity to percussion, swelling, abscess, and sinus tract, increased mobility, and radiologic assessment parameters including radiolucency in periapical or furcation area, PDL widening, and root resorption are expressed as numbers in Table 1. The differences in overall clinical and radiographic success rates between MTA and novel calcium silicate cement groups were analyzed using the Chi-square test. All statistical analyses were performed using SPSS 17.0 software (SPSS Inc., IL, Chicago, USA) at a significance level of .05.

**Table 1.** The clinical and radiographic findings in failed cases and failure rates in MTA and novel calcium silicate cement groups after 12 months.

	6 month follow-up		12 month follow-up	
	MTA	Protooth	MTA	Protooth
	(*n* = 44)	(*n* = 44)	(*n* = 41)	(*n* = 41)
Clinical evaluation				
Spontaneous pain	1	1	0	1
Swelling	0	0	0	0
Sensitivity to percussion	1	2	2	2
Sinus tract	0	0	0	1
Increased mobility	1	0	1	0
Radiographic evaluation				
Root resorption	0	0	0	0
Radiolucency in periapical or furcation area	0	0	0	0
PDL widening	0	1	0	1
Number of failures	2	3	2	3
Overall failure rate after 12 months (%)			4 (9.8%)	6 (14.6%)
Failure reasons				
6 months	MTA	1 tooth spontaneous pain and 1 tooth sensitivity to percussion and increased mobility		
	Protooth	1 tooth spontaneous pain, 1 tooth sensitivity to percussion, and 1 tooth PDL widening and sensitivity to percussion		
12 months	MTA	1 tooth sensitivity to percussion and 1 tooth sensitivity to percussion and increased mobility		
	Protooth	1 tooth spontaneous pain, 1 tooth sensitivity to percussion, and 1 tooth PDL widening, sensitivity to percussion, and sinus tract		

## Results

Forty five patients (22 boys, 23 girls, with a mean age of 7.8-years-old) were included in this clinical trial. The agreement between the examiners at the baseline session and 12-months follow-up was excellent (baseline kappa >0.9, *p* < .001 and final follow-up kappa >0.9, *p* < .001). Figure 1 illustrates the flow diagram of the study.

We excluded 41 patients due to the unexposed pulp in one of the treatment groups, where the other tooth had already received a direct pulp capping in the previous session. In addition, 11 patients were excluded in the first treatment session and 4 patients in the second treatment session because of prolonged pulpal bleeding.

At the 6-month follow-up, one patient dropped out of the study due to move to another city. We observed two failed treatments in MTA group (4.5% failure rate) and three failed treatments in novel calcium silicate cement group (6.8% failure rate), as described in Table 1. Sensitivity to percussion was the major reason for failure. Chi-square test indicated that there was no significant difference in the success rates between MTA (95.5%) and novel calcium silicate cement group (93.2%) at 6-month follow-up (risk difference = 2.3% (95% CI; −0.9% to 14.1%), *p* > .05).

At 12-month follow-up, three extra patients left the study due to lack of interest for further follow-ups. Eventually, 82 teeth in 41 patients were available for the evaluations at the end of 12 months. At 12-month follow-up, we observed an extra three teeth in the novel calcium silicate cement and two teeth in MTA group demonstrating a failed treatment (Table 1). The total of failed treatments in MTA and novel calcium silicate cement groups after 12 months were 4 and 6 cases, respectively. The Chi-square test indicated that the overall success rate between MTA (90.2%) and novel calcium silicate cement group (85.4%) was not statistically significant at the end of 12-month follow-up (risk difference = 4.9% (95% CI; −0.1% to 19.9%), *p* > .05).

## Discussion

The findings of this study suggested one-layer novel calcium silicate cement would be used for direct pulp capping in primary molars as a suitable alternative to two-layer MTA combined with overlying glass ionomer cement restored with dental amalgam after a 12-month follow-up period. Direct pulp capping is a challenging procedure in pediatric dentistry and it is indicated with precaution for primary teeth with caries [10]. The findings of our study showed comparable results in the novel calcium silicate cement group to MTA group for direct pulp capping of primary molars after both 6 and 12 months. Our findings were in agreement with the observed success rate for direct pulp capping in primary dentition using MTA [11–13].

The more prompt caries lesion progression and thinner walls of hard tissue around the pulp result in a higher risk of pulp infection in primary dentition [36]. Selecting a dental biomaterial with favorable properties including biocompatibility, antibacterial characteristics, sealing ability, and stimulation of pulp healing is crucial for a successful direct pulp capping [37,38]. However, for an optimal result, it is essential to strictly follow the inclusion criteria, aseptic procedure, and choose a proper direct pulp capping material and treatment technique.

Bacteria play a critical role in the healing of the exposed dental pulp [39]. In the current study, peripheral infected dentin was removed thoroughly and the case selection was strictly limited to exposure site less than one mm with controllable hemostasis. In an attempt to reduce the bacterial load, we constantly irrigated the cavity with sodium hypochlorite before the pulpal exposure and chlorhexidine after the pulpal exposure [40].

Calcium silicate cements as Protooth and MTA have a high calcium oxide content. During hydration, a substantial calcium hydroxide release results in a high alkaline pH [20], which provides a bacteriostatic effect for the cement [41]. Our recent preliminary findings confirm that novel calcium silicate cement compositions have comparable bacteriostatic efficacy as MTA (unpublished data). Fluoride release from the novel calcium silicate cement may further enhance the antibacterial action since the fluoride and hydroxyl ions at high pH have a synergic antibacterial effect on resistant bacteria such as *Enterococci faecalis* [42]. Moreover, fluoride release may support (re)mineralization in the adjacent tooth structure, especially in a challenging acidic environment. A long-term bacterial seal has a decisive influence on the treatment outcome of direct pulp capping [43]. Calcium silicate cements are known to provide an excellent bacterial seal [22,44], whereas calcium hydroxide-based materials might not seal efficiently in long-term because of high solubility and low bonding ability to tooth structure [45]. The advantage of using calcium silicate cements for direct pulp capping instead of calcium hydroxide materials is supported by observations in long-term studies [14,19].

The effectiveness of MTA to promote pulp healing with minimal pulp inflammation degree [46] and to stimulate dentin bridge formation after direct pulp capping has been well documented [15,47]. The current study exhibited a comparable direct pulp capping outcome of novel calcium silicate cement versus MTA, suggesting acceptable biocompatibility of the novel calcium silicate cement.

In this study, we restored the cavities with amalgam filling following direct pulp capping since it is still used as routine at the Department of Pediatric Dentistry, Tabriz University of Medical Sciences, Iran. However, Regulation (EU) 2017/852 of the European Parliament and of the Council of 17 May 2017 on mercury, and repealing Regulation (EC) No 1102/2008 suggested the termination of amalgam usage in children under the age of 15 from 1 July 2018. Resin-based dental composites would be considered for a final restoration. Our recent observations on bonding capability of the novel calcium silicate cement to different resin bonding systems support the use of resin-based filling materials including composites and compomers in such circumstances (unpublished data).

The successful clinical performance of MTA is attributed to the formation of superficial bone-like apatite layer in physiological-like solution [24]. A superficial apatite layer may introduce a suitable surface for cells to adhere and differentiate into odontoblasts [48]. Apatite formation also increases the sealing ability by forming tag-like structures inside the dentinal tubules [49] as well as increasing material bonding to the dentin [50]. Similarly, novel calcium silicate cement compositions demonstrated remarkable apatite formation ability as a function of time and fluoride content [33]. Fluoride-doped calcium silicate cements have demonstrated accelerated apatite formation and improved sealing compared with conventional calcium silicate cements [51].

The novel calcium silicate cement, as used in this study at specific powder/liquid ratio and consistency, has been developed with a fast setting time to support challenging treatments e.g. in children. The novel calcium silicate cement has a controllable fast setting time in the range of 4 to 10 min, according to the specific powder-to-liquid ratio, allowing for convenient use of the preferred consistency. The fast setting of the novel calcium silicate cement is a distinct superiority in comparison to MTA that sets in hours [30]. Ultrafast novel calcium silicate cement (Ultrafast Protooth), which sets in less than 2 min with no need to cover the cement with a wet cotton pellet, has recently been developed for mix in predosed mix-capsule. This study suggests that direct pulp capping with one-layer ultrafast novel calcium silicate cement in children, where treatment conditions allow, seem relevant for future study. We followed up the patients for 12 months to evaluate the outcome of the treatments. A recent Cochrane review has suggested a minimum of a one-year follow-up period to evaluate the successfulness of material in direct pulp capping of the primary tooth [52].

Despite a high risk of resorption in primary molars after pulp therapy, our findings suggested that direct pulp capping might be regarded as a successful conservative treatment in case of proper case selection, application of a biocompatible capping biomaterial, and an appropriate procedure. Further clinical studies with larger sample size and longer follow-ups supplemented with histological evaluations to derive a more definitive conclusion in the success of the novel calcium silicate cement in direct pulp capping of primary molars seem relevant.

The clinical and radiographic findings of the current study showed a comparable successful outcome in direct pulp capping of primary molars using one-layer novel fast-setting calcium silicate cement compared to two-layer MTA and overlying glass ionomer cement restored with amalgam after 12 months.
